# Effects of snake fungal disease (ophidiomycosis) on the skin microbiome across two major experimental scales

**DOI:** 10.1111/cobi.14411

**Published:** 2024-11-12

**Authors:** Alexander S. Romer, Matthew Grisnik, Jason W. Dallas, William Sutton, Christopher M. Murray, Rebecca H. Hardman, Tom Blanchard, Ryan J. Hanscom, Rulon W. Clark, Cody Godwin, N. Reed Alexander, Kylie C. Moe, Vincent A. Cobb, Jesse Eaker, Rob Colvin, Dustin Thames, Chris Ogle, Josh Campbell, Carlin Frost, Rachel L. Brubaker, Shawn D. Snyder, Alexander J. Rurik, Chloe E. Cummins, David W. Ludwig, Joshua L. Phillips, Donald M. Walker

**Affiliations:** ^1^ Department of Biology Middle Tennessee State University Murfreesboro Tennessee USA; ^2^ Department of Biology Coastal Carolina University Conway South Carolina USA; ^3^ Department of Agricultural and Environmental Sciences Tennessee State University Nashville Tennessee USA; ^4^ Department of Biological Sciences Southeastern Louisiana University Hammond Louisiana USA; ^5^ Center for Wildlife Health University of Tennessee Knoxville Tennessee USA; ^6^ Department of Biological Sciences University of Tennessee at Martin Martin Tennessee USA; ^7^ Department of Biology San Diego State University San Diego California USA; ^8^ Department of Natural Sciences Santa Fe College Gainesville Florida USA; ^9^ Tennessee Wildlife Resources Agency Nashville Tennessee USA; ^10^ Department of Wildlife, Fisheries and Conservation Biology University of Maine Orono Maine USA; ^11^ Department of Computer Science Middle Tennessee State University Murfreesboro Tennessee USA

**Keywords:** deep learning neural network, dysbiosis, skin microbiome, snake fungal disease, wildlife diseases, disbiosis, enfermedades de la fauna, enfermedad fúngica en serpientes, microbioma dérmico, red neural de aprendizaje profundo, **关键词**: 皮肤微生物组, 菌群失调, 蛇真菌病, 深度学习神经网络, 野生动物疾病

## Abstract

Emerging infectious diseases are increasingly recognized as a significant threat to global biodiversity conservation. Elucidating the relationship between pathogens and the host microbiome could lead to novel approaches for mitigating disease impacts. Pathogens can alter the host microbiome by inducing dysbiosis, an ecological state characterized by a reduction in bacterial alpha diversity, an increase in pathobionts, or a shift in beta diversity. We used the snake fungal disease (SFD; ophidiomycosis), system to examine how an emerging pathogen may induce dysbiosis across two experimental scales. We used quantitative polymerase chain reaction, bacterial amplicon sequencing, and a deep learning neural network to characterize the skin microbiome of free‐ranging snakes across a broad phylogenetic and spatial extent. Habitat suitability models were used to find variables associated with fungal presence on the landscape. We also conducted a laboratory study of northern watersnakes to examine temporal changes in the skin microbiome following inoculation with *Ophidiomyces ophidiicola*. Patterns characteristic of dysbiosis were found at both scales, as were nonlinear changes in alpha and alterations in beta diversity, although structural‐level and dispersion changes differed between field and laboratory contexts. The neural network was far more accurate (99.8% positive predictive value [PPV]) in predicting disease state than other analytic techniques (36.4% PPV). The genus *Pseudomonas* was characteristic of disease‐negative microbiomes, whereas, positive snakes were characterized by the pathobionts *Chryseobacterium*, *Paracoccus*, and *Sphingobacterium*. Geographic regions suitable for *O. ophidiicola* had high pathogen loads (>0.66 maximum sensitivity + specificity). We found that pathogen‐induced dysbiosis of the microbiome followed predictable trends, that disease state could be classified with neural network analyses, and that habitat suitability models predicted habitat for the SFD pathogen.

## Abstract (es)

Efectos de la enfermedad fúngica en serpientes, ofidiomicosis, sobre los microbiomas dérmicos en dos escalas experimentales importantes

**Resumen:**

Cada vez se reconoce más a las enfermedades infecciosas emergentes como una amenaza importante para la conservación mundial de la biodiversidad. Si aclaramos la relación entre los patógenos y el microbioma hospedero podríamos llegar a estrategias novedosas para mitigar el impacto de estas enfermedades. Los patógenos pueden alterar el microbioma hospedero cuando inducen la disbiosis, un estado ecológico caracterizado por la reducción de la diversidad bacteriana alfa, un incremento de los patobiontes o un cambio en la diversidad beta. Usamos la enfermedad fúngica de las serpientes, la ofidiomicosis, para analizar cómo un patógeno emergente puede inducir la disbiosis en dos escalas experimentales. Usamos la secuenciación qPCR de amplicón bacteriano y una red neural de aprendizaje profundo para caracterizar el microbioma dérmico de las serpientes silvestres de una extensa gama filogenética y espacial. Usamos modelos de idoneidad de hábitat para encontrar variables asociadas con la presencia de hongos en el paisaje. También realizamos un estudio de laboratorio sobre las culebras de agua para analizar los cambios temporales en el microbioma dérmica después de la inoculación con *Ophidiomyces ophidiicola*. Encontramos patrones característicos de la disbiosis en ambas escalas, así como cambios no lineales en la diversidad alfa y cambios en la diversidad beta, aunque los cambios a nivel estructural y en la dispersión difirieron entre los contextos del laboratorio y el silvestre. La red neural fue mucho más precisa (99.8% PPV) para predecir el estado de la enfermedad que otras técnicas de análisis (36.4% PPV). El género *Pseudomonas* fue característico de los microbiomas negativos para enfermedades, mientras que las serpientes positivas se caracterizaron por tener los patobiontes *Chryseobacterium*, *Paracoccus* y *Sphingobacterium*. Las regiones geográficas aptas para *O*. *ophidiicola* tuvieron una carga elevada de patógenos (>0.66 sensibilidad + especificidad máxima). Descubrimos que la disbiosis del microbioma inducida por patógenos sigue tendencias predecibles, que el estado de la enfermedad puede clasificarse con los análisis de red neuronal y que los modelos de idoneidad del hábitat pronosticaron el hábitat para el patógeno de la enfermedad fúngica de las serpientes.

## 跨两个主要实验尺度研究蛇真菌病对皮肤微生物组的影响

**【摘要】**人们日益认识到新发传染病已成为全球生物多样性保护的重大威胁。解析病原体与宿主微生物组之间的关系可能为减缓疾病影响提供新的策略。病原体可能通过引发菌群失调来改变宿主微生物组, 菌群失调是一种生态状态, 通常表现为细菌α多样性降低、致病菌增加或β多样性改变。本研究利用蛇真菌病(Ophidiomycosis)系统, 在两个实验尺度上探究新发病原体如何引发菌群失调。我们利用qPCR、细菌扩增子测序和深度学习神经网络, 分析了来自不同系统发育地位和空间范围的自由活动蛇类皮肤微生物组的特征。我们还使用栖息地适宜性模型来识别与真菌存在有关的景观变量。此外, 我们对北方水蛇进行了实验室研究, 以探究接种*Ophidiomyces ophidiicola*菌后皮肤微生物组的动态变化。我们在两个实验尺度上均发现了菌群失调的特征性模式, 包括α多样性和β多样性的非线性变化, 尽管结构水平和扩散变化在野外和实验室环境中有所不同。神经网络在预测疾病状态方面的准确性(99.8%的阳性预测值)远高于其他分析技术(36.4%)。未感染疾病的蛇类微生物组以假单胞菌属(*Pseudomonas*)为特征, 而患病蛇类的微生物组则以致病金黄杆菌属(*Chryseobacterium*)、副球菌属(*Paracoccus*)和鞘氨醇杆菌属(Sphingobacterium)为特征。适合*O. ophidiicola*生存的地理区域病原体负荷较高(>0.66最大灵敏度+特异性)。我们发现, 病原体引起的菌群失调具有可预测的趋势, 疾病状态可通过神经网络分析进行分类, 栖息地适宜性模型也可预测蛇真菌病病原体的适宜分布区。**【翻译:胡怡思;审校:聂永刚】**

## INTRODUCTION

In the early 2000s, global declines in snake populations were reported by multiple investigators (Lukoschek et al., 2013; Reading et al., 2010). Although the proximate cause of these declines is unknown, plausible factors include habitat degradation, climate change, and emerging pathogens (Gibbons et al., 2000). Historically, infectious diseases were considered insufficient to cause extinction events (Anderson & May, 1979; Getz & Pickering, 1983), yet disease reduces a population's size, making it more vulnerable to other mechanisms of decline (De Castro & Bolker, 2005; Fagan & Holmes, 2006). Thus, emerging infectious diseases have been identified as a leading threat to global biodiversity (Cox et al., 2022; Hoyt et al., 2021; Luedtke et al., 2023). Diseases caused by fungal pathogens are of particular concern because they are more likely to result in extinction or extirpation than other infectious agents (Fisher et al., 2020, 2012). For example, chytridiomycosis has resulted in the decline of 501 amphibian species and the extinction of 90 others (Scheele et al., 2019). Thus, the detection of a fungal pathogen *Ophidiomyces ophidiicola* in snakes, which causes snake fungal disease (SFD; ophidiomycosis), necessitates further study to mitigate potential biodiversity loss (Lorch et al., 2016). The pathogen has a broad host range (Burbrink et al., 2017; Di Nicola et al., 2022), persists in the environment (Campbell et al., 2021), and has traits that exacerbate disease impacts (Dobson, 2004; Merikanto et al., 2012), highlighting its risk to global snake populations.

Multicellular organisms are colonized by microbial assemblages distinct from the surrounding environment, referred to as the host microbiome (Berg et al., 2020; Koenig et al., 2011). Host microbiome function and composition can be altered by factors such as microtopography, host life history, and environmental heterogeneity (Grice & Segre, 2011). Perturbations to the structure and function of microbiomes that have an adverse effect on the host are referred to as dysbiosis (Petersen & Round, 2014). Dysbioses include nonmutually exclusive trends (e.g., loss of beneficial species, gain of pathobionts [Gevers et al., 2014]) and loss of microbial diversity (Petersen & Round, 2014). Zaneveld et al. (2017) expanded this definition to include stochastic changes in beta diversity, such as increased dispersion, which reflects an unstable or dysbiotic state. These processes are frequently central components of disease pathogenesis (Grice, 2014; Nakatsuji & Gallo, 2019).

An alternative explanation describing a pathogen‐altered microbiome is the adaptive microbiome hypothesis: competitive interactions in the microbiome and an immune system response act in tandem to support host health (Woodhams et al., 2023). In humans, the microbiome affects disease pathogenesis (Fitz‐Gibbon et al., 2013), and diversity often inversely correlates with disease severity (Kong et al., 2012; Paller et al., 2019). Little is known about pathogen‐induced dysbiosis (PID) in nonhuman microbiomes, especially in skin microbiome systems. The most well‐studied host–microbiome–pathogen system in wildlife is the amphibian chytrid fungus *Batrachochytrium dendrobatidis* (chytridiomycosis). Chytridiomycosis disrupts the amphibian microbiome in field populations and laboratory experiments (Jani & Briggs, 2014), correlates with an altered functional profile (Rebollar et al., 2018), and results in poor assemblage resilience (Jani et al., 2021; Woodhams et al., 2023). Similarly, *O. ophidiicola* primarily infects the skin of snakes resulting in clinical signs, including lesions, ulcerations, and granulomas (Allender et al., 2015; Lorch et al., 2015), and interacts with the host microbiome during the infection process (Allender et al., 2018; Romer et al., 2022; Walker et al., 2019). These features make it an ideal study system to test hypotheses focused on dysbiosis or an adaptive microbiome response.

Amniota (i.e., avian and nonavian reptiles, mammals, and their ancestors) share common characteristics and functions of their epidermis. During the transition to terrestrial environments, amniote skin keratinized to limit water loss, electrolyte exchange, and abrasion (Akat et al., 2022). There are several common layers of the integument in this group, including the stratum corneum (the outermost layer) and the stratum germinativum (the innermost layer; Alibardi, 2003; Wu et al., 2004). Amniotes retain a similar chemical composition of the epidermis, consisting primarily of keratin, fatty acids, and lipids (Alibardi & Toni, 2006; Baden & Maderson, 1970). Additionally, the epidermal microbiome has conserved functions in response to disease, including the production of antimicrobial peptides and regulation of host T cells (Rodrigues Hoffmann et al., 2022). Thus, SFD can serve as a model system for understanding bacterial–fungal interactions in amniote microbiomes. To facilitate this, we established the Snake Fungal Disease Working Group in 2015 to monitor the spread of *O. ophidiicola* in Tennessee (United States) and, to date, have collected ∼1000 samples across five states and 12 geographically distinct ecoregions to characterize the snake skin microbiome and its interaction with *O. ophidiicola*. The geographic, temporal, and experimental scope of our working group's sampling effort allowed us to detect broadscale ecological trends. We conducted a laboratory study to cross‐validate its results with our landscape sampling efforts.

Studies spanning differential scales in ecology allow for explicit hypothesis testing, cross‐validation of observed trends, and establishment of fundamental principles governing microbial response to fungal pathogens. In ecology, studies that pair broad landscape sampling with controlled laboratory experiments are rare. Given the complex and multifaceted dynamics of host–microbiome–pathogen systems, studies of this nature are necessary to ascertain the mechanisms and principles governing microbial responses to fungal pathogens. Accordingly, we used the SFD system to improve general understanding of disease ecology, dysbiosis, and bacterial–fungal interactions in amniote skin microbiomes across experimental scales.

Our landscape sampling featured broad spatial and temporal extents, which allowed ecological phenomena to be resolved that would not otherwise be detectable (Wiens, 1989). Given that progression of clinical signs associated with SFD generally increases over time (i.e., Lorch et al., 2015), it is likely that the microbiome is differentially affected at discrete stages of skin colonization. *Ophidiomyces ophidiicola* is likely a superior community member that exploitatively competes for nutrients, alters metabolic niche space of the microbiome, and alters host immune function (Lind et al., 2018), thus affecting microbiome composition and diversity (Allender et al., 2015). Similar patterns have been observed in other host–microbiome–pathogen systems, including in amphibians (Becker et al., 2019), fish (Zhan et al., 2022), and coral (MacKnight et al., 2021), but ours is the first study to test for dysbiosis resulting from the interaction of a skin microbiome and fungal pathogen across experimental scales in snakes.

Using the classic definitions of *dysbiosis* by Petersen and Round (2014), Gevers et al. (2014), and Zaneveld et al. (2017), we determined whether interactions between *O. ophidiicola* and the skin microbiome are generalizable across other wildlife–fungal pathogen–microbiome systems. Our objectives were to determine whether PID occurs in the skin microbiome over disease progression across two experimentally relevant scales, devise new tools to quantify pathogen‐induced changes to the skin microbiome, and model *O. ophidiicola* distribution and habitat suitability and its link to microbiome dysbiosis across the southeastern United States. Elucidating the effects of PID and the myriad of bacterial–fungal interactions that underlie this process could improve out understanding of disease ecology, improve captive breeding and bioaugmentation programs, and help inform management decisions.

## METHODS

### Field collection and live animal inoculation experimental design

Skin swab samples (*n* = 703) were collected from 2015 to 2020 in Tennessee by members of the Snake Fungal Disease Working Group using the protocol in Walker et al. (2019) (methodological details in Appendix S1). Additional samples were collected in Alabama (*n* = 10), Arkansas (*n* = 6), Georgia (*n* = 11), and Texas (*n* = 3) to increase the geographic scale of sampling, resulting in a final data set (*n* = 738 skin swabs) that encompassed 12 ecoregions (level III) (U.S. Environmental Protection Agency, 2013). In addition, we reanalyzed data from an SFD infection experiment first reported in Romer et al. (2022) (methodological details in Appendix S1). All research was conducted under Middle Tennessee State University institutional animal care and use committee 19–3001, Tennessee Wildlife Resources Agency 1547, and Tennessee Department of Environment and Conservation 2016–026.

### Molecular and bioinformatics processing of samples

We extracted DNA from skin swabs, built V4 16S rRNA libraries sequenced on a MiSeq, conducted bioinformatics in mothur v1.39.5 (Kozich et al., 2013), and performed quantitative polymerase chain reaction (qPCR) for *O. ophidiicola* quantification as in Walker et al. (2019) (methodological details in Appendix S1). Sequences were clustered into operational taxonomic units (OTUs) at 97% sequence similarity (Schloss & Westcott, 2011), because amplicon sequence variants (ASVs) artificially inflate diversity estimates by splitting bacterial genomes into separate clusters (Schloss, 2021), and negligible differences have been observed in ecological studies comparing ASVs with OTUs (Glassman & Martiny, 2018). Rare OTUs appearing 10 times or fewer in the data set were removed as an initial abundance cutoff (Cao et al., 2021). Permutation filtration for microbiome data was then used to remove rare taxa not contributing significantly to covariance structure within the OTU abundance matrix (Smirnova et al., 2019). We used the R package *decontam* to perform statistically motivated identification and removal of contaminant OTUs at a probability threshold of 0.85 (Davis et al., 2018). To normalize coverage between samples, we subsampled at 10,000 sequence reads per sample and used this data set for statistical analyses. All mothur and R code to reproduce this bioinformatics analysis is available at https://github.com/DLii‐Research/snake‐fungal‐disease. After bioinformatic processing, our final data set contained 738 samples from 19 genera of snakes encompassing 32 total species (Table 1) (GenBank BioProjects: PRJNA1114724, PRJNA 1114659).

**TABLE 1:** Summary of host snakes that had a skin swab collected from the landscape.

Host genus	Host species	Sample size	Positive snakes^a^
*Agkistrodon*	*contortrix*	66	4
	*piscivorus*	91	31
*Carphophis*	*amoenus*	15	0
*Cemophora*	*coccinea*	1	0
*Coluber*	*constrictor*	31	10
*Crotalus*	*atrox*	3	0
	*horridus* ^*^	35	15
*Diadophis*	*punctatus*	29	0
*Farancia*	*abacura*	3	0
*Heterodon*	*platirhinos*	2	1
*Lampropeltis*	*elapsoides*	1	0
	*getula*	5	4
	*nigra*	14	7
	*triangulum*	16	3
*Masticophis*	*flagellum*	1	0
*Nerodia*	*erythrogaster*	18	6
	*fasciata*	3	1
	*rhombifer*	6	0
	*sipedon*	199	77
*Opheodrys*	*aestivus*	24	0
*Pantherophis*	*guttatus*	4	1
	*obsoletus*	5	3
	*spiloides*	43	6
*Pituophis*	*melanoleucus* ^*^	1	0
*Regina*	*septemvittata*	13	4
*Sistrurus*	*miliarius* ^*^	5	0
*Storeria*	*dekayi*	33	3
	*occipitomaculata*	4	0
*Thamnophis*	*proximus*	24	9
	*sauritus*	8	1
	*sirtalis*	30	5
*Virginia*	*valeriae*	5	0
Total		738	191

*Note*: Species that are state listed as being of conservation importance are marked with an asterisk.

^a^ Positive snakes indicate those that tested positive for *Ophidiomyces ophidiicola* in quantitative polymerase chain reaction.

### Statistical analyses of field samples

Generalized additive mixed‐effects models (GAMMs) were used to model the relationship between pathogen load, as qPCR copy number, and alpha diversity (Wood, 2011). We tested for effects of pathogen load on bacterial richness and Shannon diversity. A feature selection algorithm (Boruta; Kursa & Rudnicki, 2010) was used to select random effect terms, and host genus and ecomode (Nicholson et al., 2012; Walker et al., 2019) were included as parametric terms. Univariate smooth terms included log transformation of copy number, DNA concentration of the sequenced molecular library, and day of year (by pathogen presence). Random effects included collection site, year, sequencing run, collector, and 96‐well plate columns where libraries were assembled. An Akaike information criterion (AIC) selection procedure was used to evaluate a series of distribution families and link functions (Bozdogan, 1987). A model was considered superior to another model iteration if the associated AIC value of that model was >2 below the less complex model. In instances where there was not a difference of >2, the simpler model was selected. The fit of the final models was determined by examining residual plots via the function gratia::appraise (Simpson, 2023).

The effect of disease on the microbiome was investigated as both discrete and continuous variables. We predicted ecological breakpoints resulting in alternate community states (May, 1977). Consequently, pathogen load (continuous) was divided into categories with the Jenks algorithm (Jenks & Caspall, 1971) via classIntervals::classint (Bivand, 2023). We ran this algorithm across a range of specified class numbers (*n* = 2–15) and examined the resulting goodness‐of‐fit elbow plot. We divided pathogen load into four categories defined as negative, low, moderate, and severe. Analyses of community composition revealed no difference between low and moderate disease states. Consequently, these categories were lumped into a single category called low–moderate.

Beta diversity was quantified using distance‐to‐centroid values to measure heterogeneity of microbiome composition within a group (Anderson et al., 2006). To prevent artificially inflating multivariate dispersion (distance to centroid), we excluded samples collected from outside Tennessee or from host genera sampled <10 times in a given Tennessee county (*n* = 549 samples retained) (Beck et al., 2013). Two dissimilarity metrics, Bray–Curtis and Raup–Crick, were used for this analysis. Bray–Curtis is a dissimilarity metric that places greater weight on abundant OTUs. Raup–Crick generates a null expectation for the number of shared OTUs among sites (Chase et al., 2011). This is achieved by assuming global site occupancy of taxa is equivalent to site occupancy probabilities and then accounting for sampling bias due to differences in richness between sites. Using this metric, we explicitly accounted for differences in richness on measured community dissimilarity (Chase et al., 2011). Distance‐to‐centroid values were calculated for host ecomode because this variable explained a high proportion of variation in assemblage composition. Distance‐to‐centroid values were modeled for Bray–Curtis and Raup–Crick with generalized linear mixed‐effects models (GLMMs). Disease state (categorical: negative, low–moderate, high) was included as a fixed effect. Site and sequencing run were included as random effects. The ordered beta regression family with a logistic regression link was applied to both models (Kubinec, 2023). Model fit was evaluated using DHARMa::simulateResiduals to create scaled residuals by repeatedly simulating model fit (Hartig & Lohse, 2022). An analysis of variance (ANOVA) with type II sum of squares (Fox & Weisberg, 2019) was used to determine the effect of disease state on distance to centroid values. If a significant effect was recovered, contrasts of estimated marginal means were used to perform post hoc pairwise comparisons between groups (Lenth et al., 2023).

Community composition was modeled using Bray–Curtis and Raup–Crick dissimilarity metrics. A permutation test of multivariate homogeneity of group dispersions (vegan::betadisper) was used to determine whether there were significant differences in multivariate dispersion between disease states (Oksanen et al., 2022). A Tukey honest significant difference test was used to determine what pairwise differences between groups were significant. If the larger group or groups had greater dispersion, modeling proceeded without subsetting to balance the groups because this increased the conservatism of downstream permutational multivariate ANOVAs (PERMANOVAs). We used PERMANOVAs to determine whether the multivariate centroid position varied by disease state, microbiome richness, and its interaction with pathogen detection. Permutations were stratified by site to account for spatial autocorrelation in assemblage composition (Anderson & Braak, 2003). To determine which disease states were associated with distinct assemblages, comparisons were made between disease states with a pairwise PERMANOVA via pairwiseAdonis::pairwise.adonis (Arbizu, 2017). Nonmetric multidimensional scaling (NMDS) was used to visualize community composition of the microbiome by disease state for both Bray–Curtis and Raup–Crick. Distance‐based redundancy analysis (dbRDA) was used to generate a constrained ordination of Bray–Curtis dissimilarity to illustrate the impact of pathogen load and community richness on measured community dissimilarity. Additionally, unweighted centroids were calculated for each disease state to represent the average community within a group.

### Statistical analyses of dysbiosis in vivo

To compare the field‐collected samples with a live animal experiment, we divided the original data set into subsets to focus only on the *O. ophidiicola* treatment group (*n* = 11 individuals, 83 skin swabs) for reanalysis. Previously, we used time in days after inoculation as a proxy for disease progression. However, our field data set did not have a temporal component because it consisted of point captures of unmarked individuals. Consequently, we attempted to recapitulate our analyses with pathogen load, instead of time, as a proxy for disease progression in field and experimental inoculation data sets.

A GLMM with a zero‐inflated Poisson distribution was used to model the relationship between days after inoculation and pathogen load (Brooks et al., 2017). The model included animal identity (ID) nested within capture location as a random intercept term and experimental measurement week as a separate random intercept term. A GAMM was used to model the relationship between microbiome richness and pathogen load. Measurement week, animal ID, and days after inoculation used as the slope were included as random intercept terms.

### Calculation of the dysbiosis index and bacterial–fungal interactions

Relevant taxon‐based methods to calculate microbiome dysbiosis examine taxa whose abundances are enriched or diminished due to a particular disease (Wei et al., 2021). Analysis of composition of microbiomes (ANCOM) is a differential abundance analysis used to identify features (e.g., bacterial taxa) that differ among groups while accounting for the compositional nature of microbiome data (Lin & Peddada, 2020). We used an ANCOM to determine a relevant taxon‐based dysbiosis index. The function ANCOMBC::ancombc2 was used to identify differentially abundant taxa based on both pathogen presence and fungal load. Two models were run, the first with pathogen load as the sole fixed effect term, and the second with pathogen presence as a fixed effect. Both models specified county as a random intercept term with no random slope term. Microbial genus was used as the response variable for both models. Samples were grouped by host ecomode to detect structural zeros (Lin & Peddada, 2020). A prevalence cutoff of 0.05 was utilized to remove low‐prevalence bacterial taxa (default for ANCOMBC::ancombc2). We used the approach in Gevers et al. (2014) to measure dysbiosis, as the summed abundance of taxa increase in snakes with *O. ophidiicola*, divided by the summed abundance of taxa decrease in snakes with *O. ophidiicola*. We took the natural logarithm of this ratio to generate the final dysbiosis index score for each sample. We added a pseudocount (+1) to the summed count of disease‐enriched and disease‐diminished taxa to accommodate the log transformation in the absence of a group in a sample. We used Youden's *J* statistic in OptimalCutpoints::optimal.cutpoints to select an optimal diagnostic cutoff value for this dysbiosis index based on disease state as predicted by qPCR results for fungal load.

To assess microbiome dysbiosis across disease states, data from 15 living bacterial strains in the genera *Acinetobacter* (*n* = 4), *Chryseobacterium* (*n* = 2), *Enterobacter* (*n* = 1), *Morganella* (*n* = 4), *Staphylococcus* (*n* = 3), and *Stenotrophomonas* (*n* = 1) originally isolated by Hill et al. (2018) were used to assess antifungal activity against *O. ophidiicola*. Coagulase activity, a proxy for pathogenicity, was also tested for each isolate using blood agar plates. The living strains were chosen based on sequence identity (≥97%) of taxa with high attention scores in the deep neural network model below.

### Modeling of dysbiosis with deep neural networks

We devised a novel approach in which we used a deep neural network model to identify microbial taxa important for predicting disease state (https://github.com/DLii‐Research/snake‐fungal‐disease). Unlike typical deep learning models designed for handling individual DNA sequences, we constructed an architecture capable of handling complete individual high‐throughput samples (HTSs) consisting of all sequences from a sample, aside from those identified by decontam during bioinformatics processing. The challenge with this approach is that most neural network architectures depend on the input order, whereas HTSs are unordered by nature. Thus, we assembled a model that was naturally permutation equivariant based on the set‐transformer architecture (Lee et al., 2019), making it ideal for this kind of task.

We converted the DNA sequences to 128D numeric vector representations, known as *embeddings*. We employed DNABERT (Ji et al., 2021), the state‐of‐the‐art deep learning model for embedding DNA sequences (Wang et al., 2023). This model is based on a natural language model called BERT (bidirectional encoder representations from transformers), which is designed for natural language tasks (Devlin et al., 2018). A BERT model is first trained in an unsupervised manner (pretrained) to learn contextualized embeddings by masking out part of the input sequence (text) and using the remaining input (context) to reconstruct the masked portion. After pretraining, the model can be quickly fine‐tuned to solve downstream tasks. DNABERT treats DNA sequences analogously to sentences in natural language and is able to learn high‐quality, sequence‐level, contextualized embeddings. The DNABERT model was pretrained based on the full SILVA v138.1 data set (Quast et al., 2012). However, due to the nature of HTSs, the model may be simplified by training without next‐sequence prediction, fixing the length of the sequences to 250 bp, and removing all additional tokens other than the class and mask tokens.

The final set‐transformer model takes high‐throughput subsamples as input, where each sequence in the subsample is embedded using the previous DNABERT model. Holding DNABERT's parameters constant, we pretrained this model on all of our data in a BERT fashion, in which whole sequences in the subsample were masked out with the objective of reconstructing those missing sequence embeddings. Because the order of the reconstructed sequences does not matter, a permutation‐invariant loss function was employed. For each of the embedding dimensions, the input and reconstructed sequence embeddings were sorted along a corresponding dimension before being compared. This resulted in consistent convergence, unlike other permutation‐invariant loss functions (Lee et al., 2019). After pretraining, the model was fine‐tuned to classify *O. ophidiicola* positive samples by applying a linear projection layer with a sigmoid activation to the output class token and allowing DNABERT's parameters to be updated. We performed model evaluation with 10 random leave‐out subsamples for each sample. Each subsample was evaluated independently by the deep learning model to obtain a predicted probability of that sample being *O. ophidiicola* positive. The default cutoff threshold for deciding whether a subsample is positive or negative is 0.5, but this value can be adjusted to suit the needs of the user to either reduce false positives or improve the model's recall. Once the classification prediction was made by the model for a given subsample, the attention scores were normalized and summed across the attention heads and transformer layers. Sequences with the highest attention scores were most responsible for the model's predictions, whereas sequences with the lowest scores were filtered out. As an attempt to more accurately identify the important input sequences (and their taxonomic identity), we computed the attention shift with the difference in the attention scores obtained from the fine‐tuned and the pretrained models. Analyzing the attention‐shift scores simplifies the detection of important taxa, as noise from other sequences is reduced.

### Habitat suitability modeling of *O. ophidiicola*

A combination of maximum entropy (MaxENT) (Phillips et al., 2006) and random forest (RF) algorithms (ENMeval [Kass et al., 2021] and randomForest [Liaw & Wiener, 2002]) were used to estimate habitat suitability for *O. ophidiicola*. Snakes that tested positive with qPCR were used as occurrence points. Duplicate locations, such as box trap locations with many captures, were removed to thin the data set and coupled with other standard approaches to remove spatial bias (Kramer‐Schadt et al., 2013) (Appendix S1). Overall data curation resulted in 58 SFD‐positive locations across seven ecoregions (EPA level III) in Tennessee. We selected MaxENT and RF modeling algorithms because they outperform other regression methods (Elith et al., 2006). Outputs from MaxENT and RF predicted suitability for the species of interest (range: 0–1) (detailed methods in Appendix S1). Environmental variables included data describing soil parameters from the Soil Survey Geographic Database (SSURGO) and climatic data from the WorldClim database (http://www.worldclim.org) at 30‐m resolution (Appendix S2).

Models were evaluated for fit with the area under the curve (AUC) determined through cross‐validation of five subsampled replicates. The MaxENT and RF model outputs were averaged to result in a single prediction of habitat suitability for *O. ophidiicola*. To generate a binary (presence or absence) prediction of habitat suitability for *O. ophidiicola*, we used a threshold method to dictate which raster cells were suitable following methods proposed by Liu et al. (2013). We selected the maximum sensitivity and specificity as our threshold, which maximizes the sum of the true positive rate and the true negative rate of predicted points of occurrence.

## RESULTS

### Patterns of microbiome dysbiosis in field samples

**FIGURE 1:**
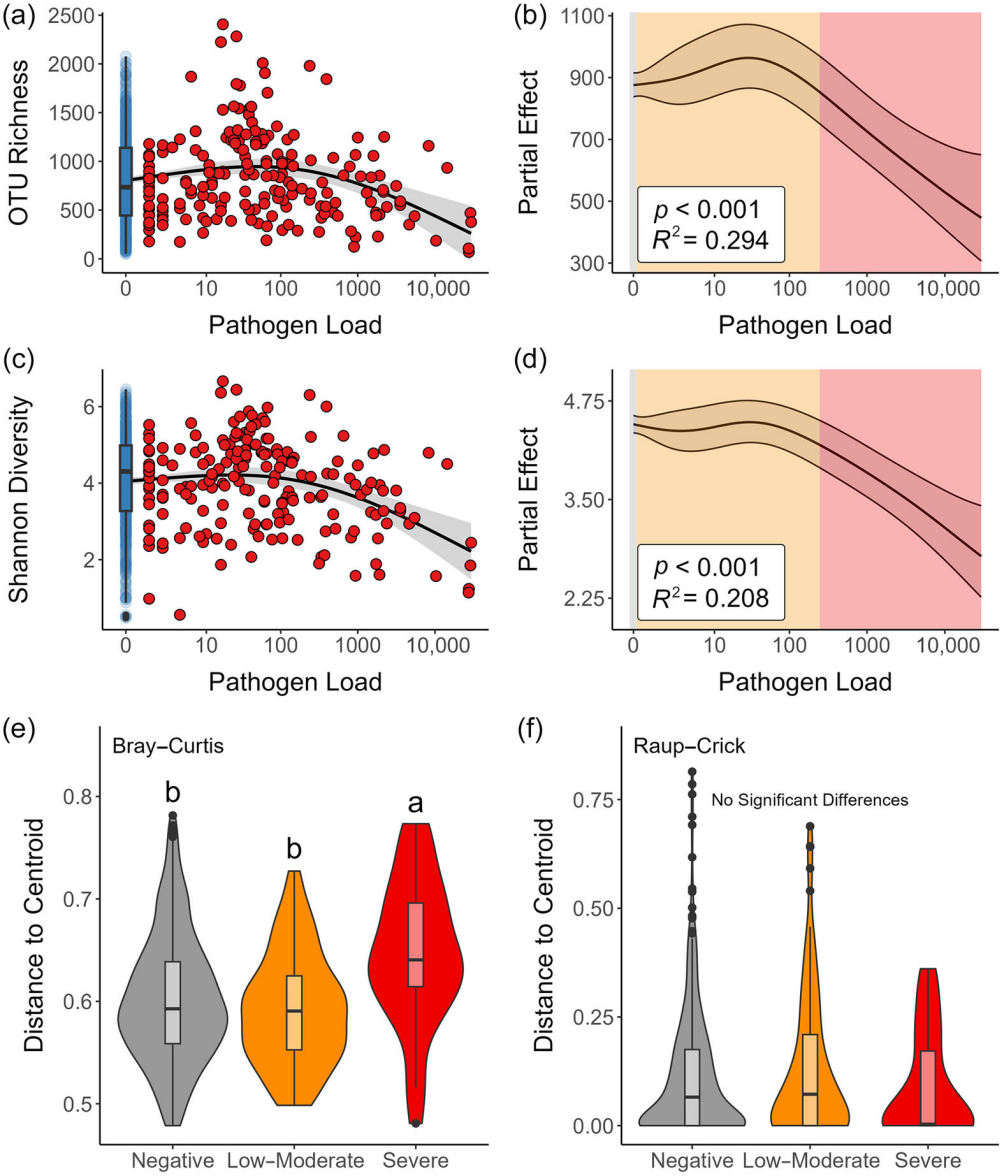
(a, c) Bacterial operational taxonomic unit (OTU) richness and Shannon diversity relative to pathogen load (quantitative polymerase chain reaction [qPCR] copy number) regression line fit via generalized additive mixed model (twoGAMM); (b, d) partial effect of pathogen load in GAMM; and (e, f) beta diversity (distance to centroid values) relative to disease state based on Bray–Curtis and Raup–Crick dissimilarity matrices. Pathogen load is a proxy for disease severity.

Table 1 summarizes collections of each snake, *O. ophidiicola* presence or absence, and conservation significance for each species sampled. Pathogen load had a significant and nonlinear effect on richness of the microbiome (GAMM, edf = 2.81, *F* = 5.34, *p* < 0.001) (Figure 1a,b). Richness of the microbiome increased as pathogen load increased, until a threshold value was reached (*n* ≈ 30 copies/reaction), after which richness declined as pathogen load increased (Figure 1a,b). Similarly, pathogen load had a significant and nonlinear effect on Shannon diversity of the microbiome (GAMM, edf = 2.91, *F* = 7.12, *p* < 0.001) (Figure 1c,d). Below ∼30 copies/reaction, Shannon diversity was not altered by changes in pathogen load. However, above this threshold, Shannon diversity declined in a similar fashion to richness (Figure 1c,d).

Disease state had a significant effect on beta diversity (measured with distance to centroid) of the microbiome when calculated using Bray–Curtis dissimilarity (GLMM, *χ* ^2^ = 19.55, *p* < 0.001) (Figure 1e). Post hoc pairwise comparisons showed significant differences between the negative and severe (EMM, *z* ratio = −4.03, *p* < 0.001) and low–moderate and severe (EMM, *z* ratio = −4.40, *p* < 0.001) disease states, as predicted by fungal load. However, we found no significant difference between negative and low–moderate disease states (EMM, *z* ratio = 1.35, *p* = 0.37). Furthermore, disease state had no significant effect on beta diversity when calculated using Raup–Crick dissimilarity (GLMM, *χ* ^2^ = 4.58, *p* = 0.10) (Figure 1f). Although SFD may result in increased heterogeneity of the microbiome, this occurred primarily through the effect of changes in richness on measured community dissimilarity.

**FIGURE 2:**
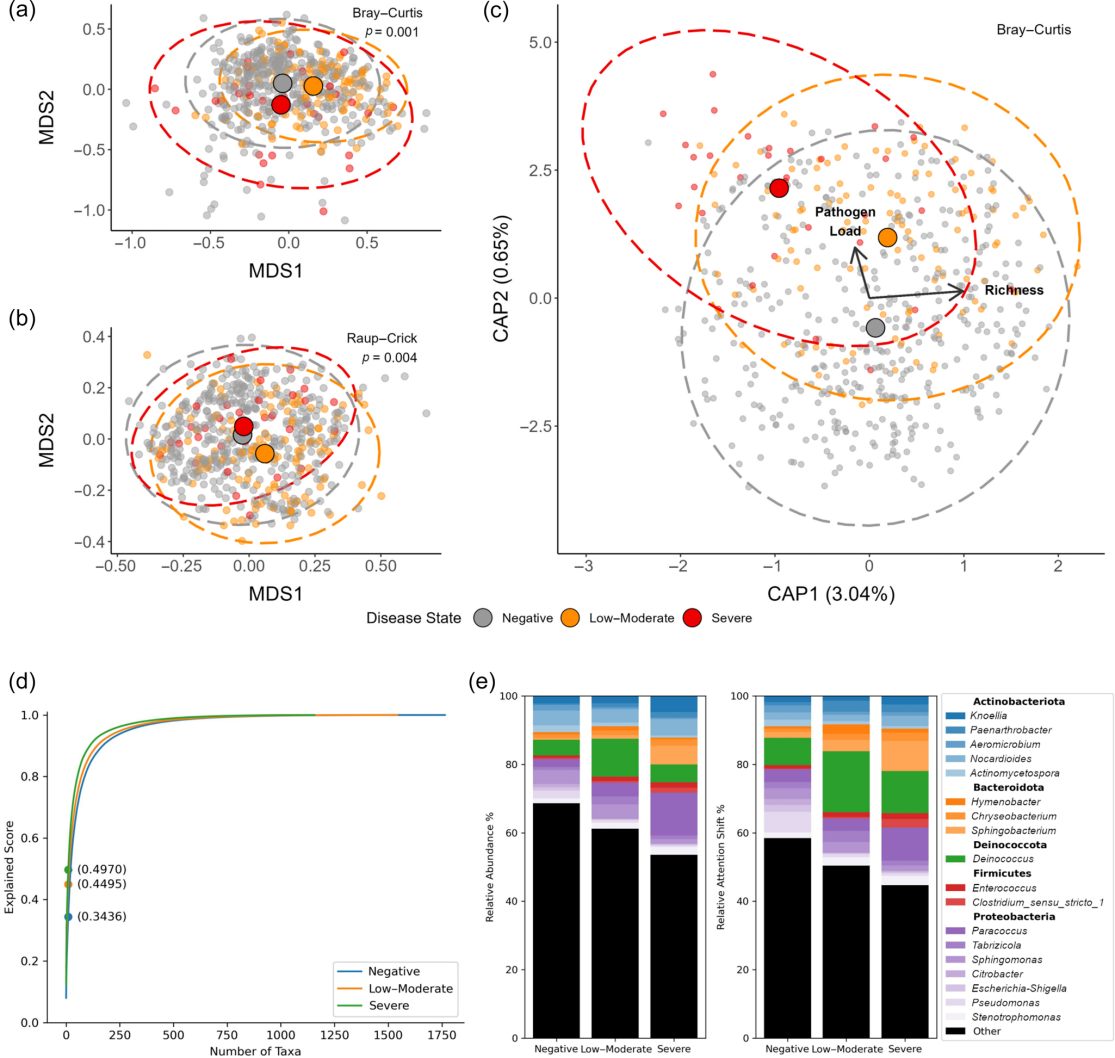
Changes in *Ophidiomyces ophidiicola* load and operational taxonomic unit richness in free‐ranging snakes: (a, b) Bray–Curtis and Raup–Crick dissimilarities as two dimensional ordinations (points, individual samples; colors, host disease state; large points, centroids in the center of 95% confidence ellipses), (c) effect of pathogen load and richness on microbiome composition based on distance‐based redundancy, (d) number of bacterial genera required for the deep neural network model to differentiate among snake disease states (points, the explained score based on the ten taxa with highest attention scores for each disease severity class in panel [e]), and (e) the relative abundance (left) and relative attention shift (right) values for bacterial taxa predictive of disease states.

Based on the Bray–Curtis metric, effects of disease state (PERMANOVA, *F* = 3.89, *p* < 0.001) and richness (PERMANOVA, *F* = 16.86, *p* < 0.001) on composition of the microbiome were significant (Figure 2a,c). However, the interaction of richness and pathogen presence had no significant effect (PERMANOVA, *F* = 1.19, *p* = 0.219). Based on the pairwise PERMANOVA, all combinations of disease state were significantly different: severe and low–moderate (*F* = 3.21, adj. *p* = 0.003), severe and negative (*F* = 3.47, adj. *p* = 0.003), negative and low–moderate (*F* = 4.14, adj. *p* = 0.003). Using the Raup–Crick metric, we again found a significant effect of disease state (PERMANOVA, *F* = 11.64, *p* = 0.002) (Figure 2b), but there was no significant effect of richness (PERMANOVA, *F* = −8.83, *p* = 0.998) or its interaction with pathogen presence (PERMANOVA, *F* = −4.46, *p* = 0.983) on microbiome composition. A pairwise PERMANOVA showed that negative and low–moderate disease states represented distinct assemblages (*F* = 19.27, adj. *p* = 0.003). However, all other pairwise combinations were not significant: severe and negative (*F* = 3.86, adj. *p* = 0.38) and severe and low–moderate (*F* = 9.06, adj. *p* = 0.063).

### Patterns of microbiome dysbiosis in the experimental inoculation study

**FIGURE 3:**
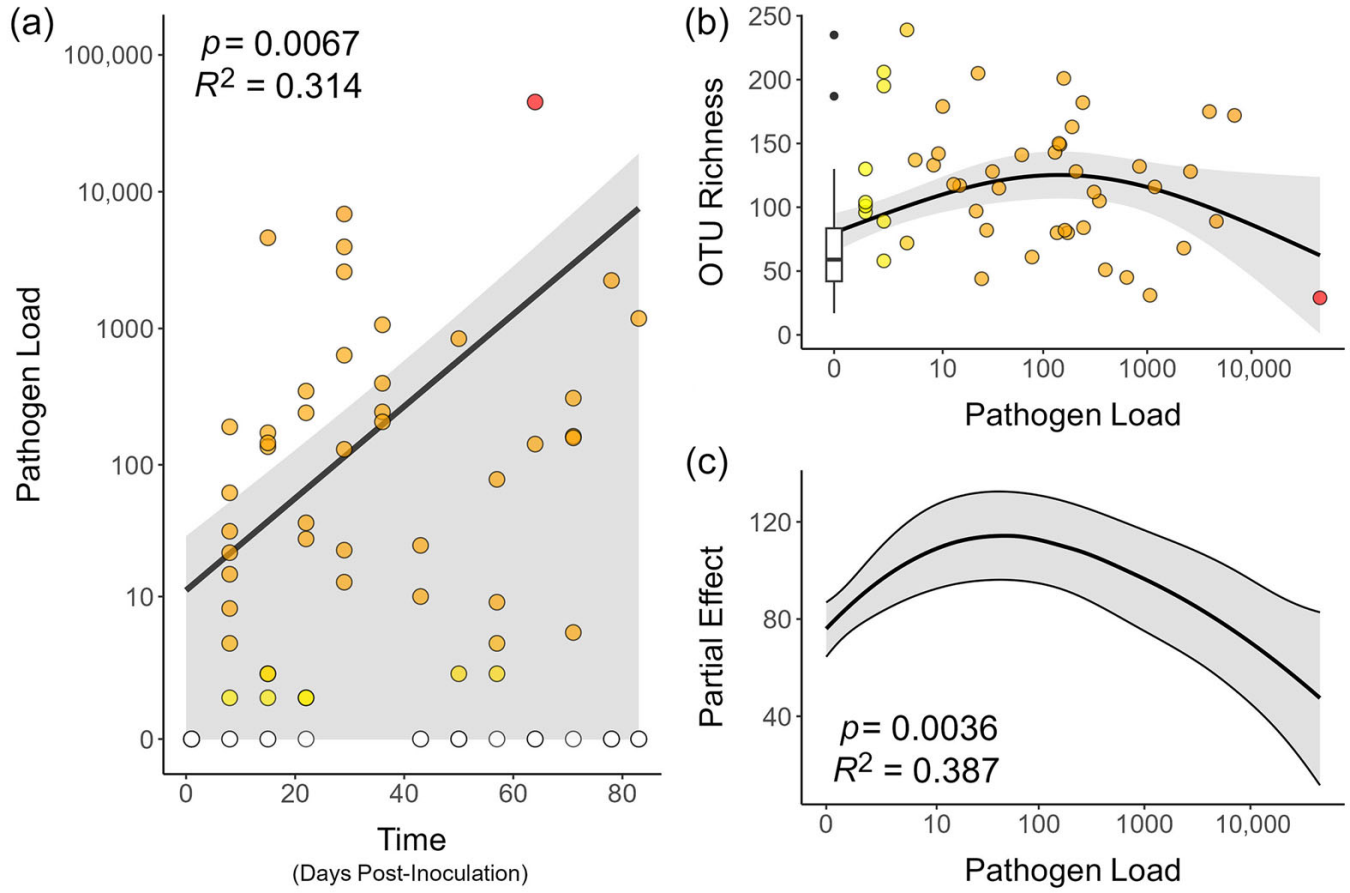
Results of reanalysis of the live snake study from Romer et al. (2022): (a) model of the effect of time (days after inoculation) on pathogen load (generalized linear mixed model with a zero‐inflated Poisson distribution), (b) operational taxonomic unit richness relative to pathogen load (quantitative polymerase chain reaction [qPCR] copy number), and (c) partial effect of pathogen load on model predictions of richness (yellow, low disease state; orange, moderate disease state; red, severe disease state; disease state indicated by qPCR).

Time measured in days after experimental inoculation had a significant positive effect on pathogen load (GLMM, *Z* = 3.34, *p* < 0.001) (Figure 3a), indicating higher pathogen load as disease progressed. Pathogen load had a significant effect on OTU richness of the microbiome (GAMM, edf = 3.18, *F* = 4.82, *p* = 0.004). Richness of the microbiome increased with pathogen load until a threshold value was reached (*n* ≈ 45 copies/reaction), after which richness declined with increasing pathogen load (Figure 3b,c). These results recapitulate the findings from the alpha diversity analyses in Romer et al. (2022); time and pathogen load followed trends of disease progression and allowed for comparisons across experimental scales (field vs. live animal).

### Detecting dysbiosis with ANCOM and deep neural networks

**FIGURE 4:**
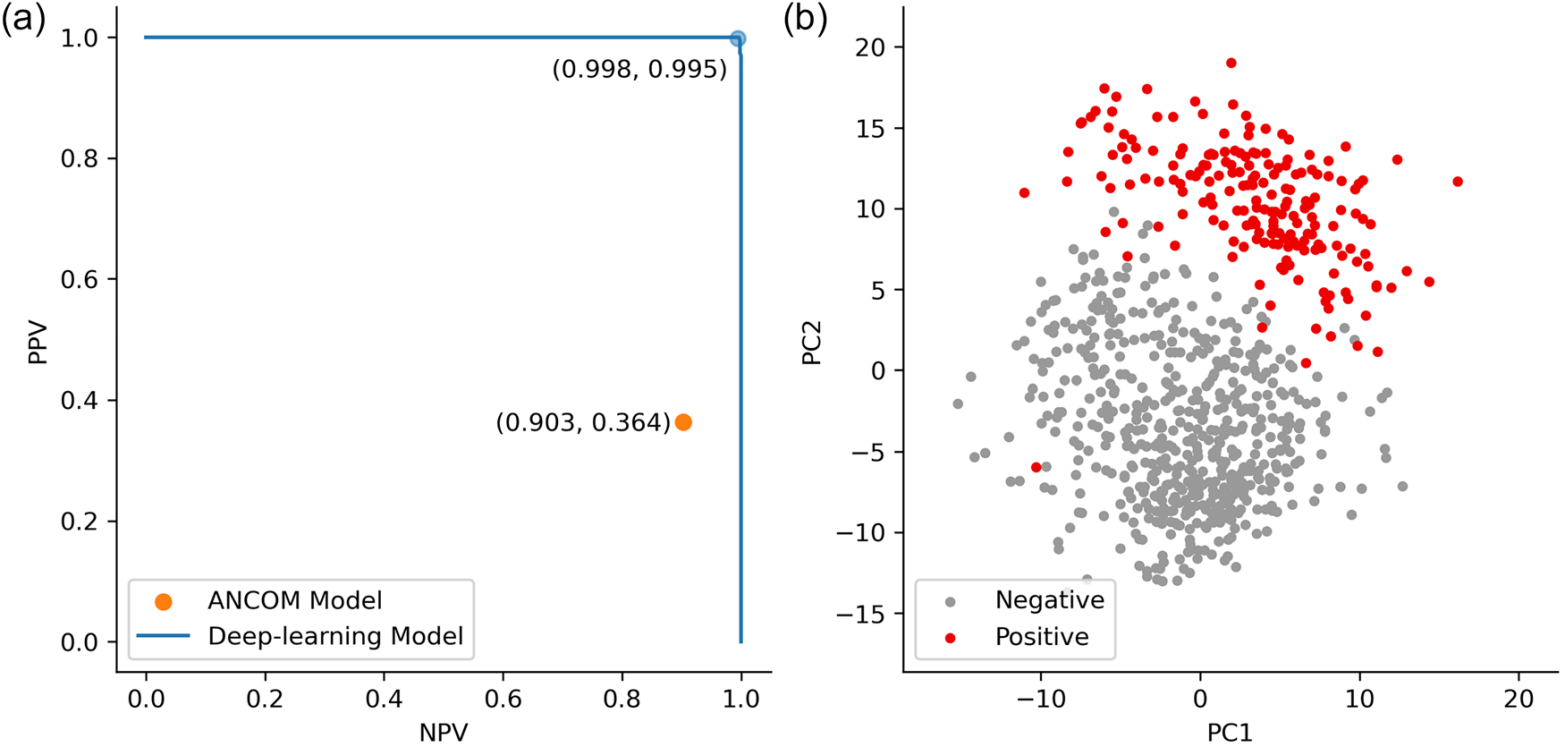
For the presence of *Ophidiomyces ophidiicola* in snakes: (a) positive predictive value and negative predictive value (PPV/NPV) of the deep‐learning and composition of microbiome (ANCOM) models (blue line, possible PPV/NPV values obtainable from the model by adjusting the prediction threshold) and (b) results of principal component analysis of the resulting learned sample embeddings (for each sample, 190 positive, 547 negative, 1000 sequences drawn at random and embedded using the deep‐learning model; principal components of embeddings project embeddings into two dimensions).

In the ANCOM model, the dysbiosis index value for differentiating samples by pathogen detection was 0.332. This metric produces a positive predictive value (PPV) of 0.364 and a negative predictive value (NPV) of 0.903. In terms of binary classification, the deep neural network model outperformed the ANCOM model (PPV = 0.998 and NPV = 0.995, respectively) (Figure 4a). An examination of the learned embeddings from the model for each sample showed a clear distinction between positive and negative samples, suggesting the important role that *O. ophidiicola* plays in structuring the microbiome (Figure 4b).

Along with classifying each of the samples by *O. ophidiicola* status, the attention shift scores identified the OTUs that best predicted disease class (Appendix S3). *Deinococcus* seemed to play more of a role in the low–moderate disease category (17.86% of attention shift) than in the negative (8.00%) or severe (12.27%) categories. For the Bacteroidota, *Hymenobacter* was attributed to low–moderate disease state (2.69%), and increased shifts for both *Chryseobacterium* (2.44%) and *Sphingobacterium* (8.85%) trended toward severe fungal loads. For the Proteobacteria, attention scores for *Paracoccus* increased from negative to severe (3.82%, 3.68%, and 9.69%). Attention scores for *Pseudomonas* were higher in negative snakes (6.05%) than in snakes with low–moderate (0.91%) and severe (0.74%) disease states, and such differences in *Pseudomonas* relative abundance were not as strong among disease states, highlighting the putative importance of *Pseudomonas* in a healthy snake microbiome.

Living strains (*n* = 15) isolated by Hill et al. (2018) scoring in the top 171 for negative, 146 for low–moderate, and 112 attention shift scores (within 90% of explained attention) (Figure 2d) for severe disease state were assessed for antifungal and coagulase activity. Two of 15 living strains showed antifungal activity (results from Hill et al., 2018) without coagulase activity, three showed only coagulase activity, one showed only antifungal activity, and three had both antifungal and coagulase activity. The remaining six strains had neither antifungal nor coagulase activity (Appendix S4).

### Habitat suitability modeling of *O. ophidiicola*

**FIGURE 5:**
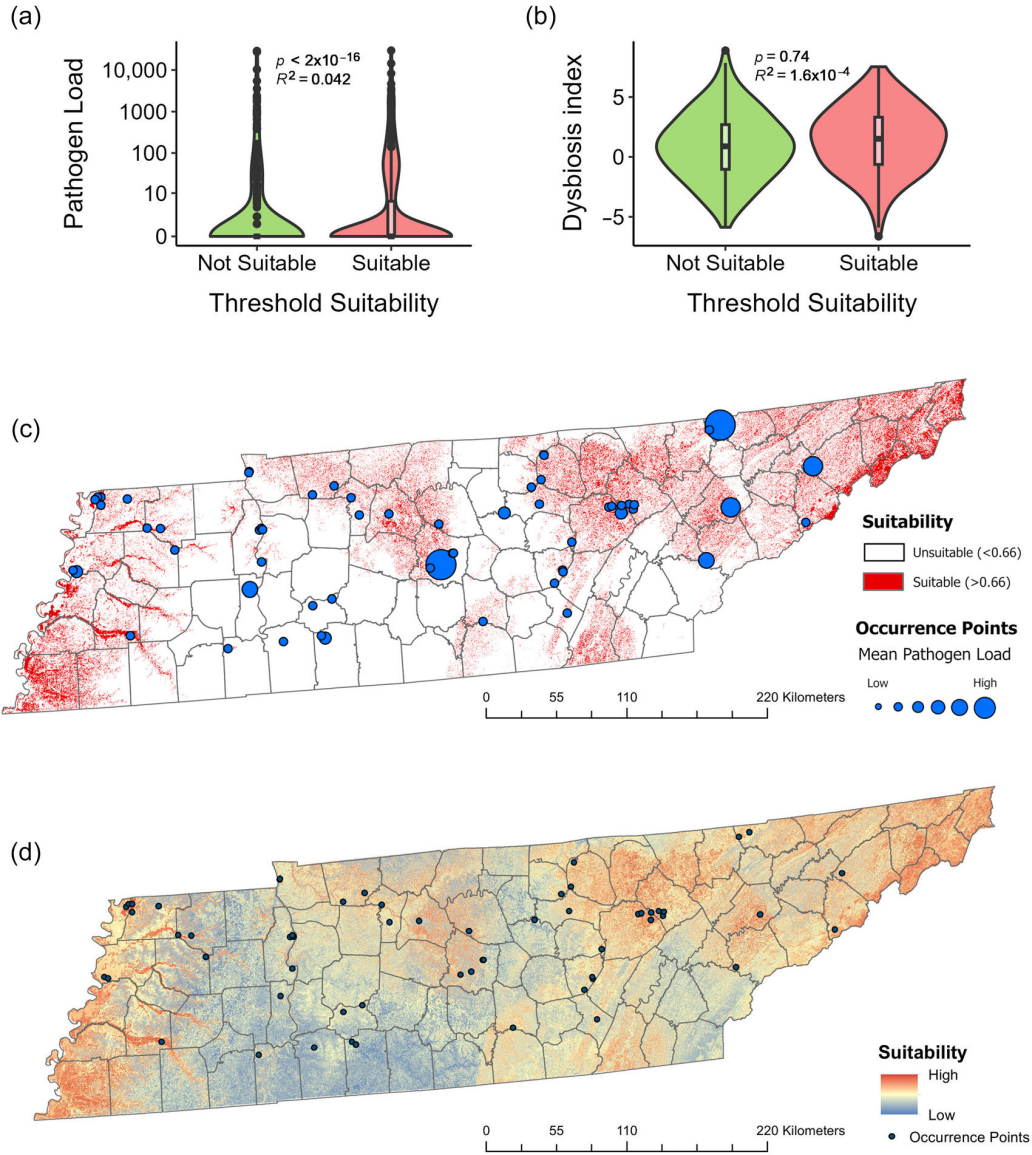
Habitat suitability modeling of *Ophidiomyces ophidiicola* across Tennessee (United States): (a) habitat for *O. ophidiicola* as predicted by species distribution modeling, (b) habitat of *O. ophidiicola* based on dysbiosis index calculated for the microbiome, (c) threshold ecological niche model for *O. ophidiicola* (red, suitability scores above 0.66 [maximum sensitivity + specificity]; occurrence points, locations of snakes testing positive with quantitative polymerase chain reaction [qPCR]; point size, mean fungal load at a site), and (d) ecological niche model for *O. ophidiicola* (the warmer the color, the higher the suitability).

**FIGURE 6:**
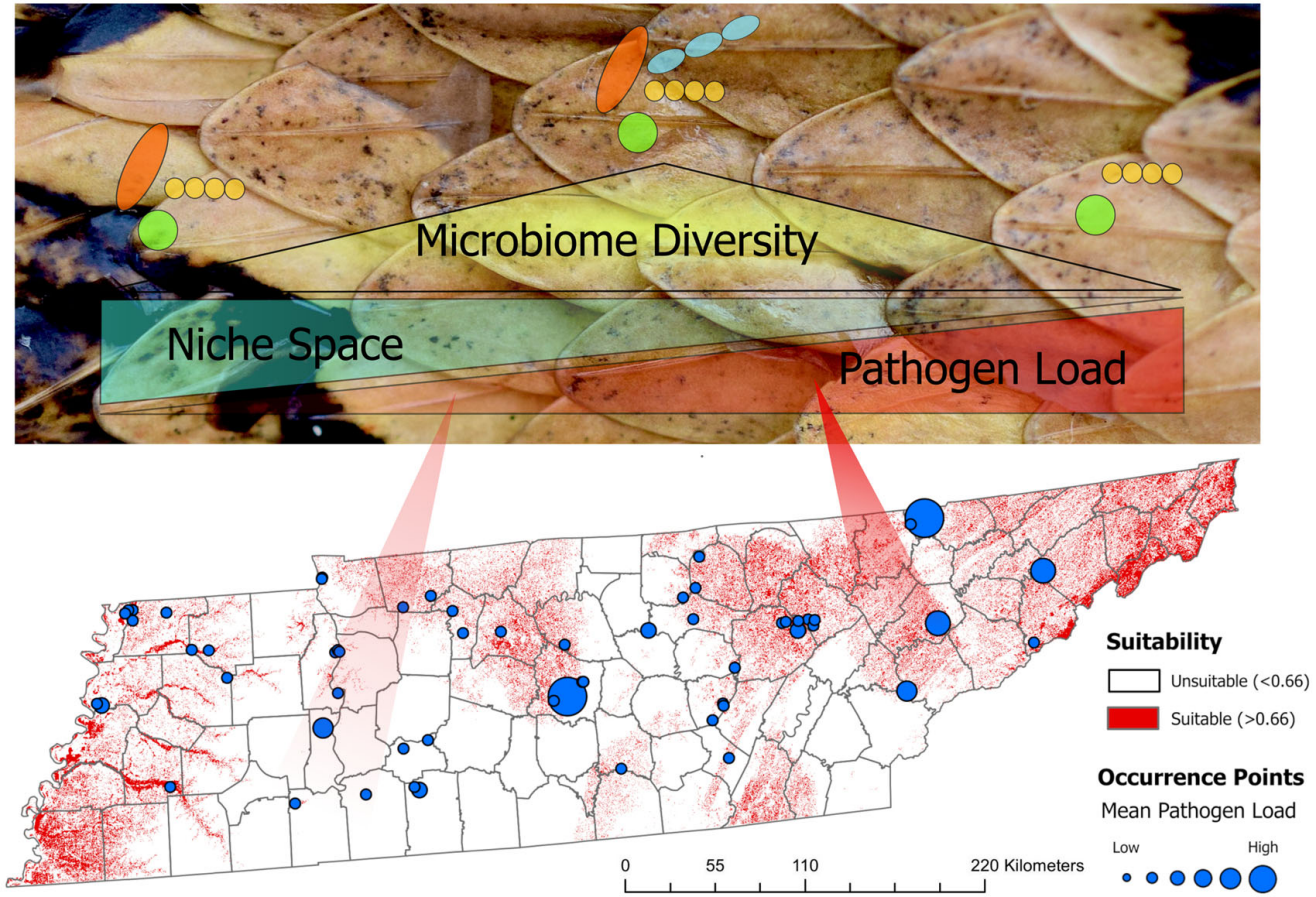
Key elements of pathogen‐induced dysbiosis by *Ophidiomyces ophidiicola*, including predictable changes in bacterial diversity (colored shapes) as pathogen load increases.

The test AUC estimates for the *O. ophidiicola* suitability models were a mean (SE) of 0.76 (0.07) for the MaxENT model and 0.71 (0.09) for the RF model (Figure 5, 6). The thresholded habitat suitability model showed a significant relationship between habitat suitability for *O. ophidiicola* and pathogen load (*p* < 0.001) (Figure 5a), but no relationship was determined between habitat suitability and the dysbiosis index (*p* = 0.74) (Figure 5b). Canopy cover (58.4%) and digital elevation (30.3%) had the greatest percent contribution to the MaxENT model. Both variables exhibited nonlinear relationships because suitability was predicted to be high at canopy cover ∼20–70% and low outside this range. Digital elevation predicted high suitability for *O. ophidiicola* at low elevation (<100 m) and at moderate‐to‐high elevation (>1000 m). For RF, annual precipitation (bio 12; 3.6%) and mean temperature of the wettest quarter (bio 8; 3.6%) had the largest contributions to the model.

## DISCUSSION

The interactions between the host microbiome and emerging fungal pathogens remain largely unexplored in wild animals and provide unique opportunities to understand disease ecology and inform biodiversity conservation practices. We used the SFD system to examine how *O. ophidiicola* influenced the skin microbiome of >700 free‐ranging snakes at the landscape and laboratory scales. We determined that disease progression resulted in changes to host–microbiome diversity; changes in pathogen load and bacterial richness alter distinct sets of microbial taxa, thereby generating unique communities along a progression of disease; our novel deep learning model was much better at predicting disease status than previous techniques; and there was a threshold relationship between habitat suitability of an area for *O. ophidiicola*, and the measured pathogen load, but not the dysbiosis index (Figure 6). Our study of an emerging fungal pathogen represents a unique approach that spans multiple scales in ecology and incorporates hypothesis testing, cross‐validation of observed trends between landscape and laboratory studies, and the development of new tools for quantifying PID.

### Patterns of microbiome dysbiosis

Bacteria and fungi share microhabitats on a host epidermis, and interactions between assemblage members influence community assembly processes and contribute to host health. A continuum of interactions from mutualism to antagonism can result in alterations to bacterial–fungal physiology, reproduction, movement, nutrition, and pathogenicity (Deveau et al., 2018). Studies that capture complex networks of interacting microorganisms across different experimental scales will improve empirical and theoretical understanding of host–microbiome–pathogen interactions and, more generally, microbiome and disease ecology. A combination of physical and chemical outcomes can occur as a result of bacterial–fungal interactions, including antibiosis, signaling and chemical exchange, adhesion with physiochemical consequences, protein secretion (Deveau et al., 2018), and host immune system regulation (Woodhams et al., 2023). Signal perception and gene expression between any or all partners are crucial in an interaction. Both fungi and bacteria respond differently depending on the interacting partners (Gkarmiri et al., 2015; Sztajer et al., 2014) and these interactions can result in microbiome dysbiosis.

We found bacterial alpha diversity in wild and captive snakes displayed a nonlinear relationship with pathogen load and declines in bacterial diversity at severe fungal loads, a pattern often associated with dysbiosis (Petersen & Round, 2014). Similarly, studies on emerging fungal pathogens, including *Pseudogymnoascus destructans* (white‐nose syndrome of bats) and *Batrachochytrium dendrobatidis* (chytrid infection of amphibians), have shown that pathogen presence is associated with declines in skin bacterial diversity (Ange‐Stark et al., 2013; Bates et al., 2018; Jani & Briggs, 2014; Schmeller et al., 2022). Previous work on the SFD–microbiome system shows that disease‐positive snakes have lower fungal alpha diversity (Allender et al., 2018), but bacterial diversity is unaffected (Allender et al., 2018; Walker et al., 2019). These studies examined SFD as categorical (presence or absence) and lacked fine‐grained details associated with pathogen load. Similar to our study, Ellison et al. (2019) found that declines in bacterial richness in the skin microbiome of frogs occur at high but not low *B. dendrobatidis* fungal loads, indicative that breakpoints may exist in the response of the microbiome to disease (D'Amario et al., 2019). The convex trends in alpha diversity may be hallmarks of fungal PID; however, a shift in skin microbiomes to a new stable state (Woodhams et al., 2023) cannot be ruled out as an alternative hypothesis.

*Ophidiomyces ophidiicola* has lipase and keratinase enzymatic activity (Allender et al., 2015) and thus likely alters the metabolic niche space available for microbes as the disease progresses. Fungal enzyme secretion and competition for nutrients or niche space may induce changes in microbiome assemblages. Our results suggest unique microbiome compositions that correlate with fungal load in negative versus low–moderate and severe disease states. Interestingly, patterns of beta dispersion (field‐collected samples) conflicted with results from the live animal experiment. Romer et al. (2022) found an increase in assemblage heterogeneity with disease progression, whereas we did not detect this trend at the landscape scale. Similar to predictions in Zaneveld et al. (2017; see their figure 2a), average structure of the microbiome at the landscape level changed in a deterministic manner (our Figure 2b), but dispersion remained the same only when controlling for differences in alpha diversity (Figure 1e,f). We observed that these effects were dependent on the distance metric used. For example, when accounting for bacterial abundance (Bray–Curtis), the skin microbiome shifted in terms of structure and dispersion. However, when bacterial presence–absence was modeled, while controlling for differences in richness (Raup–Crick), there was evidence of a minor deterministic shift in community structure but not dispersion. This difference highlights that changes in bacterial richness, particularly at the landscape scale, drive increased dispersion associated with pathogen load. Wildlife fungal pathogens are known to correlate with significant shifts in beta dispersion (Allender et al., 2018; Bates et al., 2018; Grisnik et al., 2021; Walker et al., 2019; Wilber et al., 2020). Competition for host carbon sources, such as keratin and lipids, might influence microbial interactions, disrupt assemblage structure, or influence the bacterial species present (Chang et al., 2023; Goldford et al., 2018). The importance of bacterial–fungal interactions in supporting host health or shifts to a dysbiotic state has conservation implications. Studies in natural systems or controlled live animal experiments are powerful tools with which to examine biologically meaningful patterns so as to disentangle mechanisms of microbial assembly (e.g., competition for host nutrients) in host–microbiome–pathogen systems and determine the potential for microbiomes to be used in bioaugmentation programs and conservation of biodiversity (Bletz et al., 2013; Woodhams et al., 2016).

### Deep neural networks inform microbiome analyses

Our study highlights that the use of artificial intelligence has great potential to aid in disentangling the complexities of the microbiome (Jiang et al., 2022). Deep neural networks can predict host disease status based solely on microbiome data (Grazioli et al., 2022; Lee & Rho, 2022), which is useful in determining bacterial taxa associated with host phenotypes. Because our model uses unsupervised learning of sequence data, it has broad applications for any area of study in which sets of DNA sequences are used. For example, it could be used to reanalyze raw sequence data from multiple sources in meta‐analyses to identify significant correlations that traditional microbiome analyses may struggle to detect. Furthermore, unlike typical neural networks, which are mostly a black box (Buhrmester et al., 2021), the transformer also includes the added benefit of being able to extract attention scores and weights from the internal multi‐head attention mechanism, which serve as a relative measure of the DNA sequences (a that drive predictions. Therefore, once the model determines that a given sample is *O. ophidiicola* positive, it can be used to identify which DNA sequences were used to make the prediction and their subsequent taxonomic assignment.

The normalized differences in attention scores showed the key differences in the role of bacterial taxa among disease states (Appendix S3). This was based on the intuition that there are shared interactions between healthy and diseased snakes; however, the differences might show what interactions specifically distinguish between taxa that are markers of an altered microbiome. For example, *Pseudomonas* was characteristic of disease‐negative snakes, whereas *Deinococcus* and *Hymenobacter* were associated with low–moderate disease states, and *Chryseobacterium*, *Paracoccus*, and *Sphingobacterium* were associated with severe disease states. Despite its association with the low–moderate state, *Deinococcus* likely exhibits important interactions with other taxa given that it is a major attention focus across all three conditions.

Studies of amphibian skin microbiomes show the functional importance of *Pseudomonas* species that inhibit the growth of *B. dendrobatidis* (Burkart et al., 2017; Harris et al., 2009; Lam et al., 2010; Woodhams et al., 2020). Kueneman et al. (2016) found that *Pseudomonas* species on the skin of *Anaxyrus boreas* toads decreased in response to an external stressor, and this genus was also associated with *O. ophidiicola*‐negative snakes (Allender et al., 2018), suggesting the importance of *Pseudomonas* in healthy amphibian and reptile microbiomes. *Paracoccus* has been documented as a pathogen in the microbiome of Shaw's sea snakes (*Hydrophis curtus*) (Zhong et al., 2023) and is associated with SFD (Allender et al., 2018). Likewise, *Deinococcus* and *Hymenobacter* are known indicator species of *O. ophidiicola* presence (Walker et al., 2019). *Chryseobacterium* and *Sphingobacterium* increase on frog skin in response to external stress (Becker et al., 2014; Keuneman et al., 2016) and were abundant when fungal loads on snakes were high in our study, suggesting their putative role in dysbiosis. *Chryseobacterium* also showed coagulase activity in our study, a hallmark of pathogenicity. Conversely, *Chryseobacterium* was a probiotic that interacted with yellow‐legged frog's (*Rana muscosa*) immune response to produce an antifungal peptide, suggesting its putative role in an alternative microbiome state. Hill et al. (2018) identified two species in the Proteobacteria (*Morganella* sp., *Stenotrophomonas* sp.) that inhibit *O. ophidiicola* growth in vitro. The same strain of *Morganella* demonstrated coagulase activity, but *Stenotrophomonas* did not. These genera rank among the top scoring in our deep learning model; however, *Morganella* was associated with both healthy microbiomes and with severe fungal loads, suggesting unresolved interactions among the host, microbiome, and fungal pathogen in need of further elucidation. The loss of beneficial taxa (e.g., *Pseudomonas*) and gain of pathobionts (e.g., *Paracoccus*) are characteristics of dysbiosis; however, additional work is needed to assess potential hypotheses focused on an alternative stable state of the microbiome in response to *O. ophidiicola*. Future studies could apply our deep learning model to guide selection of culture‐dependent experiments, such as synthetic microbiomes or enrichment cultures (e.g., Goldford et al., 2018; Hill et al., 2018), to address the role that bacteria with high attention scores play in bacterial–fungal interactions with *O. ophidiicola*.

### Habitat suitability modeling of *O. ophidiicola*

For wildlife diseases, ecological niche modeling can significantly aid conservation efforts by predicting regions that favor pathogen growth and disease presence. At the landscape scale, we found a significant relationship between habitat suitability of an area and pathogen load. Canopy cover and digital elevation had the largest percent contribution to our model, with intermediate levels of canopy cover (20–70%) and elevations of <100 and >1000 m being associated with high *O. ophidiicola* habitat suitability. Sites with low canopy cover are often warmer and provide snakes the opportunity to achieve high body temperatures, which have been associated with reduced *O. ophidiicola* growth in vitro (Allender et al., 2015) and in vivo (Kendall et al., 2023; McCoy et al., 2017). In contrast, high canopy cover is associated with poor thermal quality and reduced snake abundance (Blouin‐Demers & Weatherhead, 2001). Areas of low elevation in Tennessee are often associated with aquatic habitats, and previous work suggests that either *O. ophidiicola* is reliant on aquatic habitats (Lorch et al., 2016) or that semiaquatic snakes are more susceptible to *O. ophidiicola* (Blanvillain et al., 2024; McKenzie et al., 2019). We were unable to detect a relationship between habitat suitability for *O. ophidiicola* and the dysbiosis index. This suggests that although environmental factors dictate *O. ophidiicola* suitability, taxa associated with disease are either decoupled from environmental factors, or the environmental factors underlying pathogen‐dependent changes in the microbiome occur at a finer scale than those we modeled.

## CONCLUSIONS

Reptiles are among the most threatened species on Earth (Bland & Böhm, 2016; Böhm et al., 2013) due to a combination of environmental, climatic, and biotic factors. Our study represents an important step forward in understanding one such factor, emerging infectious disease, and serves as an important case study for gaining insight into this threat to global biodiversity. Our study makes major experimental, methodological, and theoretical advances in the microbiome and disease ecology of an understudied group of squamate reptiles. Most notably, our study system spans both landscape‐scale sampling and a laboratory time‐series experiment to demonstrate that bacterial–fungal interactions result in alterations to skin microbiome assemblages. These results lay the foundation for future studies of the ecology of host–microbiome–pathogen systems that can improve wildlife conservation and management efforts focused on emerging infectious diseases.

## Supporting information



Supplementary Methodology

Supplementary Methodology

Supplementary Methodology

Supplementary Methodology
