# Supplementary material for: Effects of snake fungal disease (ophidiomycosis) on the skin microbiome across two major experimental scales
Source: Conserv Biol. 2024 Nov 12;39(2):e14411. doi: 10.1111/cobi.14411 (PMC11959348; doi:10.1111/cobi.14411)
Supplement: Supplementary file 1 — Supplementary Methodology [file COBI-39-e14411-s001.docx]

**Supplementary Methodology: Molecular and bioinformatics processing of samples**

DNA was extracted from skin swabs using the Qiagen DNeasy PowerSoil HTP 96 kit, per the manufacturer’s standard protocol. Each plate included a blank well to control for kit or library preparation based contamination. The molecular presence and fungal pathogen load were detected using qPCR run in triplicate (Bohuski et al. 2015). A standard curve was generated and used to convert Ct values into log copy number for statistical analyses (detailed approach in Walker et al. 2019; Romer et al. 2022) using the formula y = -0.2893x + 10.783, where x is the average Ct for each unknown sample. Sequencing was performed according to the Illumina 16S Metagenomic Sequencing Library Preparation protocol on an Illumina MiSeq across 13 different 2 x 250bp paired-end runs. The V4 region of the 16S rDNA marker was amplified using MC Lab I-5™ 2X Hi-Fi Master Mix and dual indexed with Nextera Index Kit B using 515F and 806R as PCR primers (Kozich et al. 2013). Each library was then normalized and loaded at 4nM on the MiSeq. Raw data were demultiplexed and then a bioinformatics run was performed according to the MiSeq SOP as described in Kozich et al. (2013) with mothur v1.39.5. Sequences were clustered into operational taxonomic units (OTUs) at 97% sequence similarity (Schloss & Westcott 2011) as ASVs artificially inflate diversity estimates by splitting bacterial genomes into separate clusters (Schloss 2021) and negligible differences have been observed in ecological studies comparing ASVs to OTUs (Glassman & Martiny 2018). Rare OTUs appearing ten times or fewer in the dataset were removed as an initial abundance cutoff (Cao et al. 2021). PERFect (Permutation Filtration for Microbiome Data) was then used to remove rare taxa not contributing significantly to covariance structure within the OTU abundance matrix (Smirnova et al. 2019). Additionally, the R package *decontam* was used to perform statistically motivated identification and removal of contaminant OTUs at a probability threshold of 0.85 (Davis et al. 2018). To normalize coverage between samples, we subsampled at 10,000 sequence reads per sample, and used this dataset for statistical analyses. All mothur and R code to reproduce this bioinformatics analysis are available at https://github.com/DLii-Research/snake-fungal-disease. After bioinformatic processing, our final dataset contained 738 samples from 19 genera of snakes encompassing 32 total species (Table 1; GenBank BioProjects: PRJNA1114724, PRJNA 1114659).

**Supplementary Methodology: Field collection of samples**

Skin swab samples (n=703) were collected between 2015 - 2020 in Tennessee, USA by members of the Snake Fungal Disease Working Group (Table 1). Additional samples were collected in Alabama (n=10), Arkansas (n=6), Georgia (n=11), and Texas (n=3), to increase the geographic scale of sampling resulting in a final dataset (n = 738 skin swabs) that encompassed 12 ecoregions (level III; [U.S. Environmental Protection Agency 2013)](https://www.zotero.org/google-docs/?hlOfby). Snakes were captured using a variety of methods including visual transects, box traps, and vehicle surveys. Skin swabs were collected using a standardized protocol by rinsing of transient microbes from the snake skin with 100 mL of autoclaved (2 hours) DI water followed by 15X swab strokes over a 15 cm portion of the mid-body encompassing ventral, dorsal, and lateral surfaces (Wiens 1989)[(Walker et al. 2019)](https://www.zotero.org/google-docs/?9GLuO8). This approach allows for collection of samples with a standardized grain size, a necessity for studies quantifying patterns across space (Wiens 1989). All research was conducted under Middle Tennessee State University IACUC (IACUC 19-3001, IACUC19-3012, IACUC23-4002), Tennessee Wildlife Resource Agency permit 1547, and Tennessee Department of Environment and Conservation permit 2016-026. Swabs were stored on ice or a vehicle freezer in the field, then moved to -80°C for storage until processing.

**Supplementary Methodology: Live animal inoculation experimental design**

For this study, we reanalyzed data from an SFD infection experiment first reported in Romer et al. [(Romer et al. 2022)](https://www.zotero.org/google-docs/?9AXqfk). Briefly, field and wet lab methods are as follows: in the Spring of 2019, Common Watersnakes (*Nerodia sipedon,* n=22) were collected in Tennessee from Cheatham, Putnam, and Rutherford counties and all tested negative for *O. ophidiicola*. If an individual had clinical signs indicative of SFD or tested qPCR positive [(Bohuski et al. 2015)](https://www.zotero.org/google-docs/?V4Pb5s) then it was not included into the experiment. Using a random number generator, snakes were assigned to a sham (control; n=11) or *O. ophidiicola* inoculation (treatment; n=11) group. The inoculation procedure consisted of 15 minor skin abrasions with sandpaper to the neck, dorsal and ventral surfaces of each snake, followed by placement of a 1cm^2^ agar block of growing *O. ophidiicola* to a waterproof bandage in each body site location for 72 hours (similar to Allender et al. 2015; Lorch et al. 2015). The fungal mycelium was placed on the dermal abrasion for 72 hours to allow for *O. ophidiicola* to colonize the snake skin. For the sham treatment a sterile agar block was adhered to the abrasion sites. Due to cross-contamination of samples with SFD positive snakes, Four sham-inoculated snakes were removed from all statistical analyses resulting (n=7 sham group). To mimic a natural environment in a controlled lab setting, a microcosm with a reservoir of environmental microbes was created for the animal enclosure. Animals were maintained in 66.24L plastic storage totes with a substrate mixture of aspen bedding and soil (equal parts by weight) from each snake capture location. As *O. ophidiicola* is known to grow in the environment [(Walker et al. 2019; Campbell et al. 2021; Burger et al. 2023)](https://www.zotero.org/google-docs/?rSaWNE), all soil samples (n=22) making up the natural reservoir of microbes, were screened for *O. ophidiicola* using qPCR. The experiment began on 31 May 2019 and concluded on 21 August 2019. Throughout this period, skin swabs were collected on a weekly basis using the previously mentioned swabbing technique [(Walker et al. 2019)](https://www.zotero.org/google-docs/?w6cYNy) and processed using techniques described below. We also assessed the probability of producing a false negative with our skin swabbing technique since a previous study showed a 73% chance of *O. ophidiicola* being missed using a single swab (Hileman et al. 2018). The probability of a false negative was notably less (1st quartile - 21.6%, median - 37.5%, 3rd quartile. - 45.5%), assuming inoculated animals should have always produced a positive qPCR result reaction after week one, and dropping animals that did not produce at least three swabs due to mortality.

**Supplementary Methodology: Statistical analysis of dysbiosis in vivo**

To compare the field collected samples to the live animal experiment, we subset the dataset to the inoculated treatment group (n = 11 individuals, 83 skin swabs) for reanalysis. Previously, we used time in days post-inoculation as a proxy for disease progression. However, our field dataset does not have a temporal component, as it consists of point captures of unmarked individuals. Consequently, we attempted to recapitulate our analyses for [(Romer et al. 2022)](https://www.zotero.org/google-docs/?TM2qEK) using pathogen load (qPCR copy number) instead of time, as a proxy for disease progression in field and experimental inoculation datasets.

A generalized linear mixed model (GLMM) with a zero-inflated poisson distribution was used to model the relationship between days post-inoculation and pathogen load (Brooks et al. 2017). An AIC selection procedure was used as above to determine the most appropriate random effect terms structure. The model included animal ID nested within capture location as a random intercept term and experimental measurement week as a separate random intercept term. Model residuals were reviewed to ensure an appropriate fit. Additionally, a GAMM was used to model the relationship between microbiome richness and pathogen load. An AIC selection procedure was used to choose the most appropriate distribution family and link function (Bozdogan 1987). Measurement week, animal ID, and days post-inoculation used as the slope were included as random intercept terms. in the final model. An additional random effects term was included with animal ID as the intercept, and days post-inoculation used as the slope. The fit of the final model was determined to be appropriate by examining residual plots via the function *gratia::appraise.*

**Supplementary methodology: Habitat suitability modeling of *O. ophidiicola***

Occurrence data were filtered by thinning occurrences to allow for only one occurrence within 5km^2^. This spatial thinning reduces spatial bias caused by oversampling at well-known and accessible sites (e.g., biological research stations) as recommended in Kramer-Schadt et al. (2013).

We selected MaxENT and RF modeling algorithms because they have been shown to outperform other regression methods used for ecological niche modeling [(Elith et al. 2006)](https://www.zotero.org/google-docs/?CcJdu5).

MaxEnt is a correlative ecological niche modeling (ENM) method that combines species occurrences with environmental covariates to generate an estimate of the geographic distribution of the focal organism [(Phillips et al. 2006; Baldwin 2009)](https://www.zotero.org/google-docs/?rqy3X5). MaxENT is often favored due to model accuracy, robustness, and compatibility with ArcGIS [(Baldwin 2009)](https://www.zotero.org/google-docs/?AxbRCG). Additionally, model predictability has been shown to increase with the alteration of two parameters within MaxENT, specifically the regularization parameter and the feature class (e.g., [Warren & Seifert 2011)](https://www.zotero.org/google-docs/?e6gQTN). Therefore, combinations of four feature classes (linear, linear quadratic, linear quadratic hinge, and hinge) and four regularization parameters (0.5, 1, 2, 5) were tested. The most predictive model was selected based on a combination of AICc values and Area Under the Curve (AUC). Random Forest uses bootstrap aggregation to average regression tree outputs to predict focal organisms’ geographic distributions [(Liaw & Wiener 2002)](https://www.zotero.org/google-docs/?kV8Iwt). One of the main benefits of RF methods is the robustness of species distribution estimates from relatively few occurrence points, as well as their ability to perform better than other machine learning methods to predict relationships to under-sampled geographic locations [(Mi et al. 2017)](https://www.zotero.org/google-docs/?auVDjc). Although both modeling algorithms do not require absence locations, they do require pseudo-absence locations or background points. To generate these locations, a bias file approach was used (e.g., [Phillips & Dudík 2008)](https://www.zotero.org/google-docs/?PP1MOW), which allows for a reduction in potential bias associated with sampling effort [(Phillips et al. 2009)](https://www.zotero.org/google-docs/?qwacQL). The bias file was generated using all snake samples collected during the sampling effort. These occurrence points were then transformed into a kernel density map using a combination of the rasterize (R package *raster*; [Hijmans 2023)](https://www.zotero.org/google-docs/?xjwMY5) and the kde2d command (R package *MASS*; [Venables & Ripley 2002)](https://www.zotero.org/google-docs/?Y7xzG1), resulting in a kernel density map representing snake survey effort. Previous work has suggested that model accuracy is influenced by the ratio of occurrence points to pseudo-absence points and that this relationship is algorithm specific [(Barbet-Massin et al. 2012)](https://www.zotero.org/google-docs/?kd95qg). We followed the suggestions of [Barbet-Massin et al. (2012)](https://www.zotero.org/google-docs/?8j3Ezs) by selecting 10,000 pseudo-absence points for the MaxENT models, and an equal number of pseudo-absence points to occurrence points for the RF modeling. These pseudo-absence points were selected following the kernel density distribution, resulting in areas with high numbers of sampled snakes having a higher number of pseudo-absence points. Selecting background points using a sampling bias grid has been shown to improve model performance and reduce the influence of sampling bias [(Phillips et al. 2009)](https://www.zotero.org/google-docs/?vKD3CR).

We compiled soil environmental covariates from the Soil Survey Geographic Database (SSURGO) at 30 meter resolution and climatic data from the Worldclim database (http://www.worldclim.org) at 30-second resolution. Worldclim data includes 19 bioclimatic variables derived from global temperature and precipitation grids [(Hijmans et al. 2005)](https://www.zotero.org/google-docs/?fi5DcD). We resampled the Worldclim dataset to be at 30 m resolution using bilinear interpolation to match the resolution of the SSURGO dataset. Before analysis we tested for correlation between environmental covariates and removed those that had a Pearson's correlation coefficient of >0.75 (e.g., [Rissler & Apodaca 2007; Barrett et al. 2014)](https://www.zotero.org/google-docs/?oqC7pm), resulting in two SSURGO data layers, and four Worldclim bioclimatic data layers (Appendix S2).

**References**

Allender MC, Baker S, Wylie D, Loper D, Dreslik MJ, Phillips CA, Maddox C, Driskell EA. 2015. Development of Snake Fungal Disease after Experimental Challenge with Ophidiomyces ophiodiicola in Cottonmouths (Agkistrodon piscivorous). PLOS ONE **10**:e0140193. Public Library of Science.

[Baldwin RA. 2009. Use of Maximum Entropy Modeling in Wildlife Research. Entropy **11**:854–866. Molecular Diversity Preservation International.](https://www.zotero.org/google-docs/?UGoCDs)

[Barbet-Massin M, Jiguet F, Albert CH, Thuiller W. 2012. Selecting pseudo-absences for species distribution models: how, where and how many? Methods in Ecology and Evolution **3**:327–338.](https://www.zotero.org/google-docs/?UGoCDs)

[Barrett K, Nibbelink NP, Maerz JC. 2014. Identifying Priority Species and Conservation Opportunities Under Future Climate Scenarios: Amphibians in a Biodiversity Hotspot. Journal of Fish and Wildlife Management **5**:282–297.](https://www.zotero.org/google-docs/?UGoCDs)

Bohuski E, Lorch JM, Griffin KM, Blehert DS. 2015. TaqMan real-time polymerase chain reaction for detection of Ophidiomyces ophiodiicola, the fungus associated with snake fungal disease. BMC Veterinary Research **11**:95.

Bozdogan H. 1987. Model selection and Akaike’s Information Criterion (AIC): The general theory and its analytical extensions. Psychometrika **52**:345–370.

Brooks ME, Kristensen K, van Benthem KJ, Magnusson A, Berg CW, Nielsen A, Skaug HJ, Machler M, Bolker BM. 2017. glmmTMB balances speed and flexibility among packages for zero-inflated generalized linear mixed modeling. The R journal **9**:378–400. Technische Universitaet Wien.

Cao Q, Sun X, Rajesh K, Chalasani N, Gelow K, Katz B, Shah VH, Sanyal AJ, Smirnova E. 2021. Effects of Rare Microbiome Taxa Filtering on Statistical Analysis. Frontiers in Microbiology **11**. Frontiers. Available from https://www.frontiersin.org/journals/microbiology/articles/10.3389/fmicb.2020.607325/full (accessed June 24, 2024).

Davis NM, Proctor DM, Holmes SP, Relman DA, Callahan BJ. 2018. Simple statistical identification and removal of contaminant sequences in marker-gene and metagenomics data. Microbiome **6**:226.

[Elith J et al. 2006. Novel methods improve prediction of species’ distributions from occurrence data. Ecography **29**:129–151.](https://www.zotero.org/google-docs/?UGoCDs)

Glassman SI, Martiny JBH. 2018. Broadscale Ecological Patterns Are Robust to Use of Exact Sequence Variants versus Operational Taxonomic Units. mSphere **3**:10.1128/msphere.00148-18. American Society for Microbiology.

[Hijmans RJ. 2023. raster: Geographic Data Analysis and Modeling.](https://www.zotero.org/google-docs/?UGoCDs)

[Hijmans RJ, Cameron SE, Parra JL, Jones PG, Jarvis A. 2005. Very high resolution interpolated climate surfaces for global land areas. International Journal of Climatology **25**:1965–1978.](https://www.zotero.org/google-docs/?UGoCDs)

Hileman ET, Allender MC, Bradke DR, Faust LJ, Moore JA, Ravesi MJ, Tetzlaff SJ. 2018. Estimation of Ophidiomyces prevalence to evaluate snake fungal disease risk. The Journal of Wildlife Management **82**:173–181.

Kozich JJ, Westcott SL, Baxter NT, Highlander SK, Schloss PD. 2013. Development of a Dual-Index Sequencing Strategy and Curation Pipeline for Analyzing Amplicon Sequence Data on the MiSeq Illumina Sequencing Platform. Applied and Environmental Microbiology **79**:5112–5120. American Society for Microbiology.

[Liaw A, Wiener M. 2002. Classification and Regression by randomForest. R News **2**:18–22.](https://www.zotero.org/google-docs/?UGoCDs)

Lorch JM, Lankton J, Werner K, Falendysz EA, McCurley K, Blehert DS. 2015. Experimental Infection of Snakes with Ophidiomyces ophiodiicola Causes Pathological Changes That Typify Snake Fungal Disease. mBio **6**:10.1128/mbio.01534-15. American Society for Microbiology.

[Mi C, Huettmann F, Guo Y, Han X, Wen L. 2017. Why choose Random Forest to predict rare species distribution with few samples in large undersampled areas? Three Asian crane species models provide supporting evidence. PeerJ **5**:e2849. PeerJ Inc.](https://www.zotero.org/google-docs/?UGoCDs)

[Phillips SJ, Anderson RP, Schapire RE. 2006. Maximum entropy modeling of species geographic distributions. Ecological Modelling **190**:231–259.](https://www.zotero.org/google-docs/?UGoCDs)

[Phillips SJ, Dudík M. 2008. Modeling of species distributions with Maxent: new extensions and a comprehensive evaluation. Ecography **31**:161–175.](https://www.zotero.org/google-docs/?UGoCDs)

[Phillips SJ, Dudík M, Elith J, Graham CH, Lehmann A, Leathwick J, Ferrier S. 2009. Sample selection bias and presence-only distribution models: implications for background and pseudo-absence data. Ecological Applications **19**:181–197.](https://www.zotero.org/google-docs/?UGoCDs)

[Rissler LJ, Apodaca JJ. 2007. Adding More Ecology into Species Delimitation: Ecological Niche Models and Phylogeography Help Define Cryptic Species in the Black Salamander (Aneides flavipunctatus). Systematic Biology **56**:924–942.](https://www.zotero.org/google-docs/?UGoCDs)

Romer AS, Grinath JB, Moe KC, Walker DM. 2022. Host microbiome responses to the Snake Fungal Disease pathogen (Ophidiomyces ophidiicola) are driven by changes in microbial richness. Scientific Reports **12**:3078. Nature Publishing Group.

Schloss PD. 2021. Amplicon Sequence Variants Artificially Split Bacterial Genomes into Separate Clusters. mSphere **6**:10.1128/msphere.00191-21. American Society for Microbiology.

Schloss PD, Westcott SL. 2011. Assessing and Improving Methods Used in Operational Taxonomic Unit-Based Approaches for 16S rRNA Gene Sequence Analysis. Applied and Environmental Microbiology **77**:3219–3226. American Society for Microbiology.

Smirnova E, Huzurbazar S, Jafari F. 2019. PERFect: PERmutation Filtering test for microbiome data. Biostatistics **20**:615–631.

[Venables B, Ripley B. 2002. Modern Applied Statistics With S. Page Springer.](https://www.zotero.org/google-docs/?UGoCDs)

Walker DM, Leys JE, Grisnik M, Grajal-Puche A, Murray CM, Allender MC. 2019. Variability in snake skin microbial assemblages across spatial scales and disease states. The ISME Journal **13**:2209–2222. Nature Publishing Group.

[Warren DL, Seifert SN. 2011. Ecological niche modeling in Maxent: the importance of model complexity and the performance of model selection criteria. Ecological Applications **21**:335–342.](https://www.zotero.org/google-docs/?UGoCDs)

Weins JA. 1989. Spatial Scaling in Ecology. Functional Ecology **3**:385–397. [British Ecological Society, Wiley].
