# Supplementary material for: Effects of snake fungal disease (ophidiomycosis) on the skin microbiome across two major experimental scales
Source: Conserv Biol. 2024 Nov 12;39(2):e14411. doi: 10.1111/cobi.14411 (PMC11959348; doi:10.1111/cobi.14411)
Supplement: Supplementary file 2 — Supplementary Methodology [file COBI-39-e14411-s004.docx]

| **Covariate** | **Database** |
| --- | --- |
| Canopy Cover | SSURGO |
| DEM | SSURGO |
| Mean Diurnal Temp Range (Bio 2) | Worldclim |
| Min. Temperature of Coldest Month (Bio 6) | Worldclim |
| Mean Temperature of Wettest Quarter (Bio 8) | Worldclim |
| Annual Precipitation (Bio 12) | Worldclim |

Table 1. Environmental covariate data used in *O. ophidiicola* distribution model.
