# Supplementary material for: Effects of snake fungal disease (ophidiomycosis) on the skin microbiome across two major experimental scales
Source: Conserv Biol. 2024 Nov 12;39(2):e14411. doi: 10.1111/cobi.14411 (PMC11959348; doi:10.1111/cobi.14411)
Supplement: Supplementary file 4 — Supplementary Methodology [file COBI-39-e14411-s003.docx]

**Appendix S4:** A selection of 15 bacterial isolates that have been cultured from free-ranging snakes. The attention scores are taken from the deep neural network and shown as percentages with the ranking for that genus in the disease state given in the parentheses. Anti *Ophidiomyces ophidiicola* (*Oo*) was identified in Hill et al. (2018). Hemolysis activity was identified on sheep red blood cell agar plates after a 24 hour incubation.

| Isolate# | Bacterial Genus | Host Species | Negative Attention Score | Low-Moderate Attention Score | Severe Attention Score | Anti-*Oo* Activity | Hemolysis Activity |
| --- | --- | --- | --- | --- | --- | --- | --- |
| BR1.1 | *Chryseobacterium* | *Coluber constrictor* | 1.02 (23) | 1.85 (10) | 2.44 (8) | No | No growth |
| BR1.10 | *Chryseobacterium* | *Coluber constrictor* | 1.02 (23) | 1.85 (10) | 2.44 (8) | No | Alpha |
| BR1.11 | *Enterobacter* | *Coluber constrictor* | 0.09 (139) | 0.01 (476) | 0.06 (142) | Yes | Gamma |
| BR1.12 | *Acinetobacter* | *Coluber constrictor* | 0.81 (29) | 0.39 (47) | 1.08 (20) | No | Gamma |
| BR1.2 | *Acinetobacter* | *Coluber constrictor* | 0.81 (29) | 0.39 (47) | 1.08 (20) | No | Gamma |
| BR1.5 | *Staphylococcus* | *Coluber constrictor* | 0.99 (25) | 0.69 (27) | 0.84 (23) | No | Gamma |
| BR1.6 | *Staphylococcus* | *Coluber constrictor* | 0.99 (25) | 0.69 (27) | 0.84 (23) | No | Alpha |
| BR1.7 | *Staphylococcus* | *Coluber constrictor* | 0.99 (25) | 0.69 (27) | 0.84 (23) | No | Gamma |
| BR1.8 | *Acinetobacter* | *Coluber constrictor* | 0.81 (29) | 0.39 (47) | 1.08 (20) | No | Gamma |
| BR1.9 | *Acinetobacter* | *Coluber constrictor* | 0.81 (29) | 0.39 (47) | 1.08 (20) | No | Gamma |
| TR087-5.3 | *Morganella* | *Crotalus horridus* | 0.25 (73) | 0.08 (143) | 0.03 (216) | Yes | Alpha |
| TR087-6.4 | *Stenotrophomonas* | *Crotalus horridus* | 1.67 (13) | 2.48 (8) | 2.81 (5) | Yes | Gamma |
| TR087-7.4 | *Morganella* | *Crotalus horridus* | 0.25 (73) | 0.08 (143) | 0.03 (216) | Yes | Alpha |
| TR087-7.5 | *Morganella* | *Crotalus horridus* | 0.25 (73) | 0.08 (143) | 0.03 (216) | Yes | No growth |
| TR087-7.6 | *Morganella* | *Crotalus horridus* | 0.25 (73) | 0.08 (143) | 0.03 (216) | Yes | Alpha |

References:

Hill AJ, Leys JE, Bryan D, Erdman FM, Malone KS, Russell GN, Applegate RD, Fenton H, Niedringhaus K, Miller AN. 2018. Common cutaneous bacteria isolated from snakes inhibit growth of *Ophidiomyces ophiodiicola*. EcoHealth 15:109–120. Springer.
